# Chemical Composition and Determination of the Antibacterial Activity of Essential Oils in Liquid and Vapor Phases Extracted from Two Different Southeast Asian Herbs—*Houttuynia cordata* (Saururaceae) and *Persicaria odorata* (Polygonaceae)

**DOI:** 10.3390/molecules25102432

**Published:** 2020-05-22

**Authors:** Kristýna Řebíčková, Tomáš Bajer, David Šilha, Markéta Houdková, Karel Ventura, Petra Bajerová

**Affiliations:** 1 Department of Analytical Chemistry, Faculty of Chemical Technology, University of Pardubice, Studentská 573, 532 10 Pardubice, Czech Republic; kristyna.rebickova@student.upce.cz (K.Ř.); tomas.bajer@upce.cz (T.B.); karel.ventura@upce.cz (K.V.); 2 Department of Biological and Biochemical Sciences, Faculty of Chemical Technology, University of Pardubice, Studentská 573, 532 10 Pardubice, Czech Republic; david.silha@upce.cz; 3 Department of Crop Sciences and Agroforestry, Faculty of Tropical AgriSciences, Kamýcká 129, Czech University of Life Sciences Prague, 165 00 Prague, Czech Republic; houdkovam@ftz.czu.cz

**Keywords:** *Houttuynia cordata*, *Persicaria odorata*, essential oil, distillation, antimicrobial activity, vapor phase, volatile compounds, gas chromatography

## Abstract

Essential oils obtained via the hydrodistillation of two Asian herbs (*Houttuynia cordata* and *Persicaria odorata)* were analyzed by gas chromatography coupled to mass spectrometry (GC–MS) and gas chromatography with flame ionization detector (GC–FID). Additionally, both the liquid and vapor phase of essential oil were tested on antimicrobial activity using the broth microdilution volatilization method. Antimicrobial activity was tested on Gram-negative and Gram-positive bacteria—*Escherichia coli*, *Staphylococcus aureus*, *Pseudomonas aeruginosa*, *Enterococcus faecalis, Streptococcus pyogenes, Klebsiella pneumoniae, Seratia marcescense* and *Bacillus subtilis*. Hydrodistillation produced a yield of 0.34% (*Houttuynia cordata*) and 0.40% (*Persicaria odorata*). 41 compounds were identified in both essential oils. Essential oils contained monoterpenes and their oxidized forms, sesquiterpenes and their oxidized forms, oxidized diterpenes, derivates of phenylpropene and other groups, such as, for example, aldehydes, alcohols or fatty acids. Both essential oils were antimicrobial active in both vapor and liquid phases at least in case of one bacterium. They expressed various antimicrobial activity in the range of 128–1024 μg∙mL^−1^, 512–1024 μg∙mL^−1^ in broth and 1024 μg∙mL^−1^, 512–1024 μg∙mL^−1^ in agar, respectively. Research showed new interesting information about *P. odorata* and *H. cordata* essential oils and demonstrated that both essential oils could be possibly used in the field of natural medicine or natural food preservation.

## 1. Introduction

In recent years, many researchers have been focused on finding new antimicrobial agents that could be applied to multi-resistant microorganisms. Medicinal herbs and their products, such as essential oils (EOs), are the main source of natural remedies. They have been used since the time immemorial as the most affordable means of treating diseases. As it has been proven several times, EOs have diverse biological properties. They are bactericidal, viricidal, fungicidal, antiparasitic, antioxidant and insecticidal [1,2,3]. EOs and their components have activity against a variety of targets, particularly the membrane and cytoplasm, and, in some cases, they can completely change the morphology of the cells [4]. They contain a wide range of complex and structurally different compounds that are biologically, respectively, antimicrobially active. Antimicrobial activity is closely related to the chemical composition of EOs, functional groups and possible synergic interactions among constituents. In addition, due to the combination of these active compounds, bacteria are more difficult to develop a resistance to these compounds in comparison with commercial antimicrobials, which are usually based on one chemical substance [5]. In order to use EOs for treating various diseases caused by bacteria, it is important to understand to the relationship between their chemical composition and the potential antimicrobial activity.

EOs are volatile, and their vapors influence their antimicrobial properties. Therefore, it is necessary to sufficiently investigate the composition and properties of the vapors of essential oil. Currently, several researchers have investigated the vapor phase of the essential oil, but it deserves much wider research and consideration when examining the properties of essential oils [6,7,8,9,10,11,12]. It is assumed that active compounds in both phases are synergic and furthermore it has been shown that vapor phase is sometimes even more effective than liquid phase to some microorganisms. If biologically active molecules were present in the vapor phase, there would be, for example, the possibility of using EOs for the inhalation and treatment of respiratory diseases [8,9]. Furthermore, there would be the possibility of using these properties of EOs to produce various food packaging materials that protect food against the spread of microorganisms and extend their shelf life [13,14,15].

Traditionally, agar dilution, broth dilution or broth microdilution methods have been used to measure the minimal inhibitory concentrations of antimicrobial agents against microorganisms [16,17]. Unfortunately, these methods have been limited to measuring the minimal inhibitory concentrations (MICs) of antimicrobials only in the liquid phase. In the last few years, several methods and the modification-testing MICs of antimicrobials in the vapor phase were developed. Usually, the disc volatilization method with various modifications is used [10,11,12,17,18].

Two Southeast Asian herbs (*Houttuynia cordata* and *Persicaria odorata*), used mainly in the traditional Chinese medicine and as a spice in Asian cuisine, have been chosen for this study. These two herbs have been chosen for their known health benefits from Chinese medicine and for their geographical occurrence. Moreover, there is a lack of scientific literature about *Houttuynia cordata* [19,20,21,22,23,24] and *Persicaria odorata* [25,26] EOs. Therefore, they were examined in more detail. *Houttuynia cordata*, also known as a fish mint, belongs to a family *Saururaceae*. It is a flowering perennial herb native to Southeast Asia and it grows in moist shady places [27]. EOs from *H. cordata* have a fishy scent and show a variety of biological activities, such as antimicrobial, antiviral, anti-inflammatory, anticancer and insect repellent [28]. According to the previous reports, the liquid phase of EOs from *H. cordata* has inhibitory effects against *Pseudomonas aeruginosa*, *Escherichia coli*, *Bacillus cereus*, *Bacillus subtilis*, *Vibrio cholerae* and *Staphylococcus aureus* [21,29]. The dominant volatile compounds are houttuynin, myrcene, decanal, *cis*-ocimene and bornyl acetate [27,28,30]. *Persicaria odorata*, also known as a Vietnamese coriander, belongs to a family *Polygonaceae*. It is a tender perennial herb native to Southeast Asia and it grows in wet environments with a rich, moist soil shady places [25]. EOs isolated from *P. odorata* have a very strong coriander odor and show antimicrobial, anti-inflammatory, antitumor and antioxidant activity. According to the previous reports, the liquid phase of essential oil from *P. odorata* inhibits *Salmonella choleraesuis* [31], *Enterococcus faecalis*, *Enterococcus faecium, Staphylococcus epidermidis* and *Staphylococcus aureus* [32]. The most abundant volatile compounds are dodecanal, decanal, 3-hexanal, 2-hexanal, β-caryophyllene and α-humulene [25,26,33,34].

Due to the growing demand for natural products and in light of the prevalence of pharmaceuticals, antioxidants or food additives in the food preservation process, it is necessary to find new sources for developing these products and to specify their properties. Plant-derived essential oils have received significant attention in this field. The aim of this study was to find herbs used both in the cuisine and natural medicine and determine their EOs composition and especially their antimicrobial efficiency in both vapor and liquid phase. In this study were two EOs obtained by hydrodistillation and thereafter analyzed by standard techniques GC–MS and GC–FID. Antimicrobial activity was determined by the modern, recently developed method of Houdkova et al. [35] called the broth microdilution volatilization method. It is a simple and rapid simultaneous determination of the antibacterial potential of plant volatile compounds in the liquid and the vapor phase at different concentrations [35].

## 2. Results and Discussion

### 2.1. Extraction Yield and Chemical Composition

Hydrodistillation in the Clevenger-type apparatus of *Houttuynia cordata* produced a pale-yellow liquid with a fishy scent. The essential oil content of distilled aerial parts of dried plant was 0.34%. The extraction yield is higher in comparison with those previously published by R. S. Verma et al. [24] who only achieved a yield of 0.06–0.14%. A total of 41 compounds were identified that made up 90.6% of the essential oil composition (Table 1). The essential oil contained a higher amount of terpenoid compounds (75.5%), followed by non-terpenoid compounds (15.1%), such as derivates of phenylpropene, aldehydes, ketones, esters and fatty acids. The major group of substances was monoterpenes with a content of 59.4%, followed by the group of other compounds with a content of 14.8%, oxidized monoterpenes with a content of 7.2% and sesquiterpenes with a content of 6.6%. Other groups were oxidized sesquiterpenes and derivates of phenylpropene. Major compounds of the essential oil were myrcene (51.6%), 2-undecanone (6.7%), tridecan-2-one (6.1%), *cis-*β-ocimene (5.7%), geranyl acetate (3.1%), bornyl acetate (2.9%) and *cis-*caryophyllene (2.6%). The other compounds were present at less than 2%. These results are similar with the results of previous reports [19,22,24]. Only a few fluctuations from other reports were found and are probably attributed to the origin of the plant samples or different extraction method. For the characteristic fishy scent and flavoring of *H. cordata* essential oils is responsible compound houttuynin (decanoyl acetaldehyde). This compound was not identified in our essential oil due to its instability. It is usual that decanoyl acetaldehyde is during the process of distillation easily oxidized into 2-undecanone [24]. This compound had the second highest concentration in our essential oil. Therefore, the amount of 2-undecanone is the primary indicator for the quality of *Houttuynia cordata* essential oil [23,24].

Hydrodistillation in Clevenger-type apparatus of *Persicaria odorata* produced a deep yellow liquid with a strong spicy coriander-like aroma. Due to its aroma, it is also called Vietnamese coriander [25]. The essential oil content of distilled aerial parts of dried plant was 0.41%. The extraction yield is lower in comparison with those previously published by A. A. Almarie et al. [33] who achieved a yield of 0.64%. A total of 41 compounds were identified that made up 90.4% of the essential oil composition. In comparison with other reports, we identified more compounds [38,39]. N. X. Dung et al. [38] used steam distillation for the isolation of essential oils and they identified 28 compounds and the most abundant were β-caryophyllene, dodecanal and caryophyllene oxide. M. V. Hunter et al. [39] only identified 17 compounds using steam distillation as an extraction technique for the isolation of essential oil from *P. odorata*, where the most abundant compounds were α-humulene, decanal and dodecanal. Our essential oil contained a higher amount of non-terpenoid compounds (72.7%) followed by terpenoid compounds (17.7%). Carbonyls and alcohols, especially C10 and C12, made up 68.8% of essential oil composition, followed by the group of sesquiterpenes with a content of 11.5% and oxidized sesquiterpenes with a content of 5.7%. Other groups (monoterpenes, oxidized monoterpenes and oxidized diterpenes) made up less than 1% of essential oil constitution. The major compounds of the essential oil were *n*-dodecanal (37.1%), *n*-decanal (18.1%), 1-decanol (5.4%), 1-dodecanol (4.8%), α-humulene (4.5%), *cis*-caryophyllene (3.9%) and *n*-undecane (2.5%). The other compounds were present at less than 2%. These results in relative percent content are similar with the results of previous reports. It can clearly be seen that the essential oil from *Persicaria odorata* is rich in C10 and C12 carbonyls. Dodecanal and decanal are the main compounds of *Persicaria odorata* essential oil in all previous published reports and ours [25,33,38,39].

When comparing both EOs, it was found that they have only 12 common compounds out of 41 but they vary in the percent content. The essential oil from *Hottuynia cordata* contained much more monoterpenes and monoterpenoids, where the most abundant compound was myrcene (51%) that was not found in the essential oil from the *P. odorata*. On the other hand, the essential oil from *Persicaria odorata* contained more sesquiterpenes, sesquipterpenoids and especially aldehydes, where *n*-dodecanal (37.1%) was the dominant compound compared to *H. cordata* essential oil, where it was only 0.02%. In general, the composition of both essential oils is different. The results are adequate because both herbs are neither from the same genus nor family, so their similarity in composition was not expected. They were selected for this study according to their similar use and same geographical occurrence.

### 2.2. Antimicrobial Activity

The antimicrobial activity of *H. cordata* and *P. odorata* essential oils is reported in Table 2. Both EOs showed antimicrobial efficiency but in different concentrations. *H. cordata* and *P. odorata* essential oil expressed various antimicrobial activity in the range of 128–1024 μg∙mL^−1^, 512–1024 μg∙mL^−1^ in broth and 1024 μg∙mL^−1^, 512–1024 μg∙mL^−1^ in agar, respectively. In the liquid phase, the lowest MIC was showed for *H. cordata* (128 μg∙mL^−1^) against *E. faecalis* and for *P. odorata* (512 μg∙mL^−1^) against *S. pyogenes*, *E. faecalis* and *B. subtilis*. In the vapor phase, the lowest MIC was observed for *H. cordata* (1024 μg∙mL^−1^) against *E. faecalis* and *E. coli* and for *P. odorata* (512 μg∙mL^−1^) against *E. coli*. There are observable differences between the efficiency of the vapor and liquid phases of observed essential oils (EOs). In most cases, the higher MIC reached the liquid phase, except in the case of EOs from *P. odorata* on *E. coli*, where the vapor phase was twice as effective as the liquid phase.

In general, Gram-negative bacteria are more resistant to EOs than Gram-positive bacteria [40]. This is supported by our results because, as it is shown in Table 2, Gram-positive bacteria were more sensitive to tested EOs in comparison with Gram-negative bacteria. It is possible that active compounds in EOs can more easily break important bonds (peptidoglycan) in the cell wall structure of Gram-positive bacteria. The structure of the Gram-positive bacteria cell wall allows hydrophobic molecules to easily penetrate the cells and act on both the cell wall and within the cytoplasm. After the cell wall is broken, the reactive constituents of the essential oil can penetrate the interior of the cell and further damage its DNA. The other fact is that phenolic compounds, which are also present in the EOs, generally show antimicrobial activity against Gram-positive bacteria. On the other hand, the cell wall of Gram-negative bacteria is far more complex, and it is, among other reasons, why they are more resistant to biologically active compounds (EOs) [4].

The most abundant compound in *H. cordata* essential oil is myrcene. Myrcene has an antimicrobial activity and moreover enhances the activity of antibiotics [41]. EOs with a high content of myrcene have a positive effect on urinary and genital infections [42,43]. These infections may be caused among others by *E. coli* and *E. faecalis*; therefore, we could assume that EOs from *H. cordata* will affect them, which has been confirmed in this study. The most abundant compound in *P. odorata* essential oil was α-humulene, which is known for its anti-inflammatory effect. It is well known that EOs with α-humulene are natural antimicrobial agents [44,45,46]. Pichette et al. [46] have tested the antimicrobial activity of α-humulene against *E. coli* and *S. aureus* using the microdillution method. α-humulene exhibited an MIC of 2.6 μg∙mL^−1^ against *S. aureus* and an MIC of more than 20 μg∙mL^−1^ against *E. coli*. Jang et al. [45] have tested the antimicrobial activity of α-humulene against *B. fragilis* and obtained MIC of 0.5 μg∙mL^−1^. The high content of α-humulene could be the reason why *P. odorata* EOs inhibited the growth of six from eight tested bacteria. On the other hand, there is one possible disadvantage when using these oils orally or internally, which is possible irritation or allergy caused by *cis*-caryophyllene, which both EOs contains [47]. It would be necessary to further examine the negative effect of each compound in the essential oil on the human body before using it for treating illness.

As far as authors know, there are no previous reports about *Persicaria odorata* and *Houttuynia cordata* essential oils and its antimicrobial activity in the vapor phase, so it is not possible to further compare those results with other publications. However, there are some reports about testing the liquid phase of EOs from *Houttuynia cordata*. Verma et al. [24] tested the antimicrobial activity of the essential oil from *Houttuynia cordata* against four bacteria (*Staphylococcus aures*, *Streptococcus mutans*, *Mycobacterium smegmatis* and *Enteroccocus faecalis*). Their essential oil exhibited MIC in the range of 0.52–1.04 μL∙mL^−1^. Ji et al. [20] performed a disc diffusion test to determine antimicrobial activity of *H. cordata* essential oil against *Bacillus subtilis*, *Escherichia coli* and *Staphylococcus aureus*; unfortunately, the disc diffusion test is only a screening method, which is not possible to compare with MIC. Lu et al. [22] tested the antimicrobial activity of *H. cordata* EOs against *Staphylococcus aureus* and *Sarcina ureae* using the broth and agar dilution method. Their reached minimal inhibitory concentration was in the range of 0.5–1.0 μL∙mL^−1^.

## 3. Materials and Methods

### 3.1. Plant Material

Approximately 120 g of fresh Chinese herbs (*H. cordata* and *P. odorata*) was purchased in a local Vietnamese market (TTTM Sapa, Prague, Czech Republic). Each sample was air dried in a dark room at the laboratory’s temperature. Prior to the distillation, both herbs, including leaves and stems, were crushed into smaller pieces.

### 3.2. Essential Oil Isolation

Essential oils were obtained by hydrodistillation using Clevenger-type apparatus. The EOs was prepared as follows: 26.7 g (*H. cordata*) or 18.4 g (*P. odorata*) of dried herb was weighted into a 2000 mL distillation flask, 1000 mL of water was added and the EOs was distilled for 4 h. The essential oil was then separated from hydrosol and stored in sealed dark-glass vials at 4 °C until the analysis.

### 3.3. Bacterial Strains and Culture Media

Four Gram-negative and four Gram-positive bacteria that caused respiratory infections, including upper and lower airway diseases, were chosen. Standard strains were used for the experiment: *Escherichia coli* CCM 3954, *Pseudomonas aeruginosa* CCM 3955, *Klebsiella pneumoniae* NPK12, *Serratia marcescens* CCM 303, *Staphylococcus aureus* CCM 4223, *Enterococcus faecalis* CCM 4224, *Streptococcus pyogenes* NPK01 and *Bacillus subtilis* CCM 2215. All standard strains were purchased from Czech Collection of Microorganisms (CCM), Brno, Czech Republic. Bacteria were incubated at 37 °C for 24 h. Prior to the experiment, bacterial suspensions with turbidity according to the McFarland scale were prepared corresponding to the grade 0.5 (~1.5 × 10^8^ CFU·mL^−1^). The cultivation and assay media were Mueller-Hinton (agar and broth; Himedia, India). The pH of the broth was adjusted to a final value of 7.6 using Trizma base and hydrochloric acid (both Himedia, India). Ampicilin (St. Louis, MO, USA) was used as a positive antibiotic control.

### 3.4. Antimicrobial Activity Assay

The antimicrobial activity of the liquid and vapor phase of Eos was determined by the broth microdilution volatilization method [35]. The experiments were carried out in 96-well microtiter plates with one well volume of 400 µL. The test is designed for the testing of 6 essential oils in total. For this study, we tested only 2 essential oils, and different samples were in other wells. The plates were covered with wooden plates and clamped to prevent vapor phase leakage. The edge wells were left blank to avoid the edge effect. First, essential oil samples were prepared as follows: approximately 2 µL of EOs was added to corresponding amount of dimethyl sulfoxide (DMSO) at a concentration of 1%, then further diluted in the corresponding broth to initial concentration. Then, the antibiotic was prepared at an initial concentration of 4 µg∙mL^−1^. In the first part of the experiment, 30 µL of agar was pipetted onto the plate lid and inoculated with 5 µL of bacterial suspension for vapor phase testing. In the second part (liquid phase assay), 100 µL of buffered Mueller-Hinton broth was pipetted into the wells. Each well had a final volume of 100 µL. Seven two-fold diluted concentrations of samples starting at a concentration of 1024 µg∙mL^−1^ were prepared for each essential oil in one row. A positive and negative control of bacterial growth was prepared in the first two columns. In the last column, 6 two-fold diluted concentrations of antibiotic starting at 4 µg∙mL^−1^ were prepared. Finally, all wells except the negative control were inoculated with 5 µL of bacterial suspension. Plates were closed, fixed and incubated at 37 °C for 24 h. After incubation, minimal inhibitory concentrations of EOs were evaluated by the visual assessment of bacterial growth after the coloring of a metabolically active bacterial colony with thiazolyl blue tetrazolium bromide dye (MTT; Sigma Aldrich, Prague, Czech Republic). A total of 25 µL of 600 µg∙mL^−1^ dye was applied to the lid and each well of the plate and equilibrated for 10 min. The color changed from yellow (dead cells) to purple (live cells). Thereafter, the MICs were recorded. All experiments were performed in triplicate in three independent experiments. The results were expressed as the median of minimal inhibition concentration of the antimicrobial agent values.

### 3.5. GC–MS Analysis

The GC–MS analysis of samples was carried out by using a Gas Chromatograph GC 2010 coupled to a Mass Selective Detector GCMS-QP2010 Plus (both Shimadzu, Kyoto, Japan) and Combi Pal Autosampler (CTC Analytics, AG, Zwingen, Swizerland) on a capillary column SLB-5ms Supelco (30 m × 0.25 mm, 2.5 μm film thickness; Bellefonte, PA, USA). The carrier gas was Helium 5.0 (Linde, Prague, Czech Republic) with a constant flow of 30 cm·s^−1^. The oven temperature program was set at an initial temperature of 40 °C for 3 min, then heated up to 250 °C at 2 °C·min^−1^ and held at 250 °C for 10 min. The injector and detector temperatures were set at 200 °C. The mass spectrometry detector was operated under electron ionization mode at ionization energy of 70 eV when ions with *m*/*z* 33–500 were scanned. A total of 1 µL of diluted essential oil (200 times, *n*-hexane) was injected with a split ratio 1:50. The experimental results of retention indices were calculated relative to C8–C33 *n*-alkanes in concentrations of 100–200 µg·mL^−1^, dissolved in *n*-hexane (Restek, Bellefonte, PA, USA). The calculation was performed according to the van den Dool and Kratz method, and the results were further compared to published data [36,37]. Compounds were identified by comparing their mass spectra with mass spectra of several standards (Table 1) and commercial mass spectral databases NIST’14 Mass Spectral Library and FFNSC 2 GC/MS Library Release 2.0 (Flavor and Fragrance Natural and Synthetic Compounds Library) and further checked out by manual mass spectra evaluation.

### 3.6. GC–FID Analysis

The GC–FID analysis of samples was carried out by using a Gas Chromatograph GC 2010 with a flame ionization detector (Shimadzu, Kyoto, Japan) and Autosampler Combi Pal (CTC Analytics, AG, Zwingen, Swizerland) on a capillary column SLB-5ms Supelco (30 m × 0.25 mm, 2.5 μm film thickness; Bellefonte, PA, USA). The GC–FID conditions were the same as in case of GC–MS analysis. The injector temperature was set at 200 °C and the detector temperature was set at 260 °C. A total of 1 µL of diluted essential oil (200 times, *n*-hexane) was injected with a split ratio 1:50. As in the case of GC–MS, experimental retention indices were calculated and compared to published data.

## 4. Conclusions

This study shows new interesting knowledge about EOs distilled from two Asian herbs—*Persicaria odorata* and *Houttuynia cordata*. The chemical composition of EOs corresponds to previous studies with a minor deviation that may be caused by agronomic factors, sample storage or sample preparation and other factors. Both EOs showed antimicrobial activity in different concentrations to different bacteria. Due to great antibacterial activity, along with the composition of Eos, we see a great potential for future usages of these oils, such as natural antimicrobials or food preservatives. As far as we know, we were the first to describe the antimicrobial properties of those EOs in both vapor and liquid phases on eight selected bacteria. Furthermore, it is necessary to study the possible cytotoxicity of these oils. The disadvantage is that both oils contain *cis*-caryophyllene that causes allergic reactions and skin irritation. It would be necessary to find the balance in concentrations of beneficial antimicrobial active compounds and potentially toxic compounds.

## Figures and Tables

**Table 1:** Chemical composition of essential oils from *Houttuynia cordata* and *Persicaria odorata*.

Compound	CAS Number	Identification ^1^	Retention Index		Peak Area [%]	
			Observed	Published ^2^	*H. cordata*	*P. odorata*
**Monoterpenes**						
α-pinene	80-56-8	MS, RI	930	933	0.53	-
camphene	79-92-5	MS, RI	946	953	0.36	-
β-pinene	127-91-3	MS, RI	974	978	0.45	-
myrcene	123-35-3	MS, RI, Std	993	991	51.64	-
limonene	138-86-3	MS, RI, Std	1027	1030	0.49	-
*cis-*β-ocimene	3338-55-4	MS, RI	1036	1040	5.72	-
*trans-*β-ocimene	3779-61-1	MS, RI	1046	1046	0.18	-
7*-epi*-sesquithujene	159407-35-9	MS, RI	1387	1387	-	0.02
Sum [%]					59.37	0.02
**Oxidized monoterpenes**						
perillene	539-52-6	MS, RI	1097	1098	0.51	-
linalool	78-70-6	MS, RI, Std	1100	1101	0.28	-
myroxide	33281-83-3	MS, RI	1131	1129	0.01	-
isoborneol	10385-78-1	MS, RI	1169	1165	0.08	-
α-terpineol	98-55-5	MS, RI, Std	1193	1195	0.06	-
β-cyclocitral	432-25-7	MS, RI	1218	1219	-	0.04
*trans*-geraniol	102-24-1	MS, RI	1251	1255	0.2	-
bornyl acetate	92618-89-8	MS, RI	1283	1285	2.85	-
neryl acetate	141-12-8	MS, RI	1358	1365	0.12	-
geranyl acetate	105-87-3	MS, RI, Std	1378	1383	3.11	-
Sum [%]					7.22	0.04
**Sesquiterpenes**						
*cis*-caryophyllene	13877-93-5	MS, RI	1419	1424	2.58	3.88
*trans-*α-bergamotene	13474-59-4	MS, RI	1431	1432	0.1	0.25
isogermacrene D	317819-80-0	MS, RI	1441	1447	-	0.08
*trans*-caryophyllene	87-44-5	MS, RI	1452	1451	0.99	-
α-humulene	6753-98-6	MS, RI, Std	1455	1454	-	4.50
γ-gurjunene	22567-17- 5	MS, RI	1474	1476	-	0.30
selina-4.11-diene	17627-30-4	MS, RI	1476	1482	1.13	0.23
α-curcumene	644-30-4	MS, RI	1482	1480	-	0.23
β-selinene	17066-67-0	MS, RI	1487	1491	-	0.40
valencene	4630-07-3	MS, RI	1490	1492	0.67	0.04
β-bisabolene	4891-79-6	MS, RI	1508	1508	-	0.07
β-curcumene	72345-84-7	MS, RI	1510	1511	-	0.08
7*-epi*-α-selinene	123123-37-5	MS, RI	1517	1518	0.54	0.52
*trans*-calamenene	73209-42-4	MS, RI	1519	1527	0.24	-
*cis*-sesquisabinene hydrate	58319-05-4	MS, RI	1543	1544	-	0.96
Sum [%]					6.25	11.54
**Oxidized sesquiterpenes**						
β-elemene	33880-83-0	MS, RI	1387	1390	0.13	-
ishwarane	26620-70-2	MS, RI	1465	1468	0.24	-
α-farnesene	502-61-4	MS, RI	1503	1504	0.18	-
β-nerolidol	40716-66-3	MS, RI	1560	1561	0.76	0.53
spathulenol	72203-24-8	MS, RI	1575	1576	0.37	-
caryophyllene oxide	1139-30-6	MS, RI	1580	1587	0.99	1.42
humulene epoxide II	19888-34-7	MS, RI	1608	1613	-	1.09
caryophylla-4(12).8(13)-dien-5-ol	19431-80-2	MS, RI	1632	1636	-	0.69
*epi*-β-bisabolol	235421-59-7	MS, RI	1669	1675	-	0.34
α-bisabolol	515-69-5	MS, RI	1686	1688	-	0.05
*trans-*α-bergamotol	88034-74-6	MS, RI	1688	1688	-	0.05
drimenol	19078-37-6	MS, RI	1768	1769	-	1.24
drimenin	2326-89-8	MS, RI	1944	1944	-	0.30
Sum [%]					2.67	5.71
**Oxidized diterpenes**						
phytone	502-69-2	MS, RI	1840	1841	-	0.38
Sum [%]					0	0.38
**Derivates of phenylpropene**						
methyl eugenol	93-15-2	MS, RI	1401	1403	0.25	-
Sum [%]					0.25	0
**Others**						
6-methyl-hept-5-en-2-one	110-93-0	MS, RI	985	986	0.05	-
2-pentyl-furan	3777-69-3	MS, RI	993	991	-	0.06
6-methyl-Hept-5-en-2-ol	1569-60-4	MS, RI	998	995	-	0.03
*n*-undecane	1120-21-4	MS, RI	1101	1100	-	2.52
*n*-nonanal	124-19-6	MS, RI	1104	1107	0.12	0.26
1-nonanol	143-08-8	MS, RI	1172	1169	0.33	0.35
*n*-decanal	112-31-2	MS, RI, Std	1208	1208	0.15	18.4
1-decanol	112-30-1	MS, RI	1276	1278	-	5.37
2-undecanone	112-12-9	MS, RI	1293	1294	6.67	-
*n*-undecanal	112-44-7	MS, RI	1307	1309	-	1.37
1-undecanol	112-42-5	MS, RI	1377	1379	-	1.16
2-dodecanone	6175-49-1	MS, RI	1393	1393	0.07	-
*n*-dodecanal	112-54-9	MS, RI, Std	1411	1410	0.02	37.08
1-dodecanol	112-53-8	MS, RI, Std	1477	1476	-	4.81
tridecan-2-one	593-08-8	MS, RI	1496	1495	6.06	-
*n*-dodecanoic acid	143-07-7	MS, RI	1569	1570	0.70	-
*n*-tetradecanal	124-5-4	MS, RI	1612	1614	-	0.26
intermedeol	6168-59-8	MS, RI	1661	1668	-	0.13
2-pentadecanone	2345-28-0	MS, RI	1696	1697	0.21	-
*n*-hexadecanoic acid	57-10-3	MS, RI	1963	1968	0.45	0.44
linoleoyl chloride	7459-33-8	MS, RI	2135	2139	-	0.16
*n*-dodecenylsuccinic anhydride	1978-11-1	MS, RI	2159	2159	-	0.29
Sum [%]					14.83	72.69
**Total peak area [%]**					90.59	90.38

^1^ MS—mass spectra, RI—retention index, Std—analytical standard; ^2^ Data published in database ofNational Institute of Standards and Technology (NIST) [36] and Adams [37].

**Table 2:** Antimicrobial activity of tested essential oils and antibiotic ampicillin against Gram-negative and Gram-positive bacteria.

Bacterium	Sample/Growth/MIC (μg∙mL^−1^)					
	*Houttuynia cordata*		*Persicaria odorata*		*Ampicillin*	
	Agar	Broth	Agar	Broth	Agar	Broth
**Gram negative**						
*Escherichia coli*	1024	512	512	1024	>4	0.50
*Pseudomonas aeruginosa*	>1024	>1024	>1024	1024	>4	1.00
*Klebsiella pneumoniae*	>1024	1024	>1024	1024	>4	>4.00
*Serratia marcescens*	>1024	1024	>1024	>1024	>4	4.00
**Gram positive**						
*Staphylococcus aureus*	>1024	1024	>1024	>1024	>4	0.50
*Enterococcus faecalis*	1024	128	>1024	512	>4	0.25
*Streptococcus pyogenes*	>1024	512	1024	512	>4	1.00
*Bacillus subtilis*	>1024	>1024	>1024	512	>4	2.00
